# Midazolam infusions for therapeutic management of pediatric refractory status epilepticus: a systematic review

**DOI:** 10.3389/fped.2025.1507325

**Published:** 2025-04-14

**Authors:** K. Taneille Johnson, Ammar AlZadjali, Dawoud Al Nasseri, Jessie Cunningham, Kazuhiro Shoya, Cecil D. Hahn, John Basmaji, Nicole K. McKinnon

**Affiliations:** ^1^ Department of Paediatrics, Schulich School of Medicine and Dentistry, Western University, London, ON, Canada; ^2^ Department of Pediatrics, Sohar Hospital, Sohar, Oman; ^3^ Department of Pediatrics, Ibri Hospital, Ibri, Oman; ^4^ Health Sciences Library, The Hospital for Sick Children, Toronto, ON, Canada; ^5^ Department of Critical Care Medicine, Sakakibara Heart Institute, Tokyo, Japan; ^6^ Department of Paediatrics, Division of Neurology, The Hospital for Sick Children, Toronto, ON, Canada; ^7^ Program in Neurosciences and Mental Health, Hospital for Sick Children Research Institute, Toronto, ON, Canada; ^8^ Department of Paediatrics, Temerty School of Medicine, University of Toronto, Toronto, ON, Canada; ^9^ Division of Critical Care, London Health Sciences, Western University, London, ON, Canada; ^10^ Department of Physiology, Temerty School of Medicine, University of Toronto, Toronto, ON, Canada; ^11^ Department of Critical Care Medicine, The Hospital for Sick Children, Toronto, ON, Canada

**Keywords:** refractory status epilepticus, treatment, midazolam, therapeutic window, intensive care units, pediatric

## Abstract

**Objective:**

We aim to determine the optimal dosing of midazolam continuous intravenous infusions for the treatment of pediatric refractory status epilepticus (RSE).

**Data sources:**

We searched Medline ALL, Embase, Embase Classic, Cochrane CENTRAL, and Web of Science in March 2023 and again in February 2024.

**Study selection:**

Randomized and non-randomized studies involving pediatric patients who received continuous midazolam for the treatment of RSE were eligible. Two authors independently conducted screening, full-text review, and data extraction. All methods followed PRISMA reporting guidelines. A narrative data synthesis was performed due to data heterogeneity.

**Data extraction and synthesis:**

Nineteen studies (448 patients) proved eligible; 3 were randomized control trials, while 16 were non-randomized studies. All studies had concerns regarding the risk of bias. Overall, midazolam aborted seizures in 363/448 (81%) participants, with mean effective doses of 1.7–13.0 μg/kg/min (0.17–0.78 mg/kg/h). The remaining 85 participants (19%) who did not achieve seizure cessation received maximum doses of 1.7–32.0 μg/kg/min (0.17–1.92 mg/kg/h) prior to transitioning to another agent. Only 4 studies specified that boluses were given with each titration. Twelve studies reported that seizure cessation occurred at a mean time of 1.4–546.0 min (range 0–720 min) after midazolam initiation. In 8 of these studies, effective midazolam doses clustered at 2.0–5.0 μg/kg/min (0.12–0.30 mg/kg/h), with seizure cessation occurring within 10–70 min in 204/221 (92%) participants. Treatment-associated adverse events included intubation in 42/221 (19%) and hypotension requiring fluids or no intervention in 18/221 (8%). The studies did not differentiate between intubations performed as part of the study protocol or prior to midazolam infusion initiation nor did they specify whether hypotension was related to the co-administration of phenytoin or phenobarbital.

**Conclusion:**

Data supporting midazolam continuous infusion dosing are limited and heterogeneous. Our findings suggest a potential therapeutic window at rates of 2.0–5.0 μg/kg/min (0.12–0.30 mg/kg/h), with limited adverse risks. Earlier seizure cessation may be achieved by targeting this therapeutic window by starting treatment with higher doses than the typically used 1.0 μg/kg/min (0.06 mg/kg/h) or by rapidly escalating the dose.

**Systematic Review Registration:** PROSPERO, identifier CRD42023413038.

## Introduction

Approximately 25% of children presenting with seizures will develop refractory status epilepticus (RSE), defined as ongoing seizures despite the administration of an appropriately dosed benzodiazepine and a second-line anti-seizure medication with a different mechanism of action (1–3). Despite over 4 decades of research, we have not yet determined the optimal management strategy for pediatric RSE.

**Figure 1:**
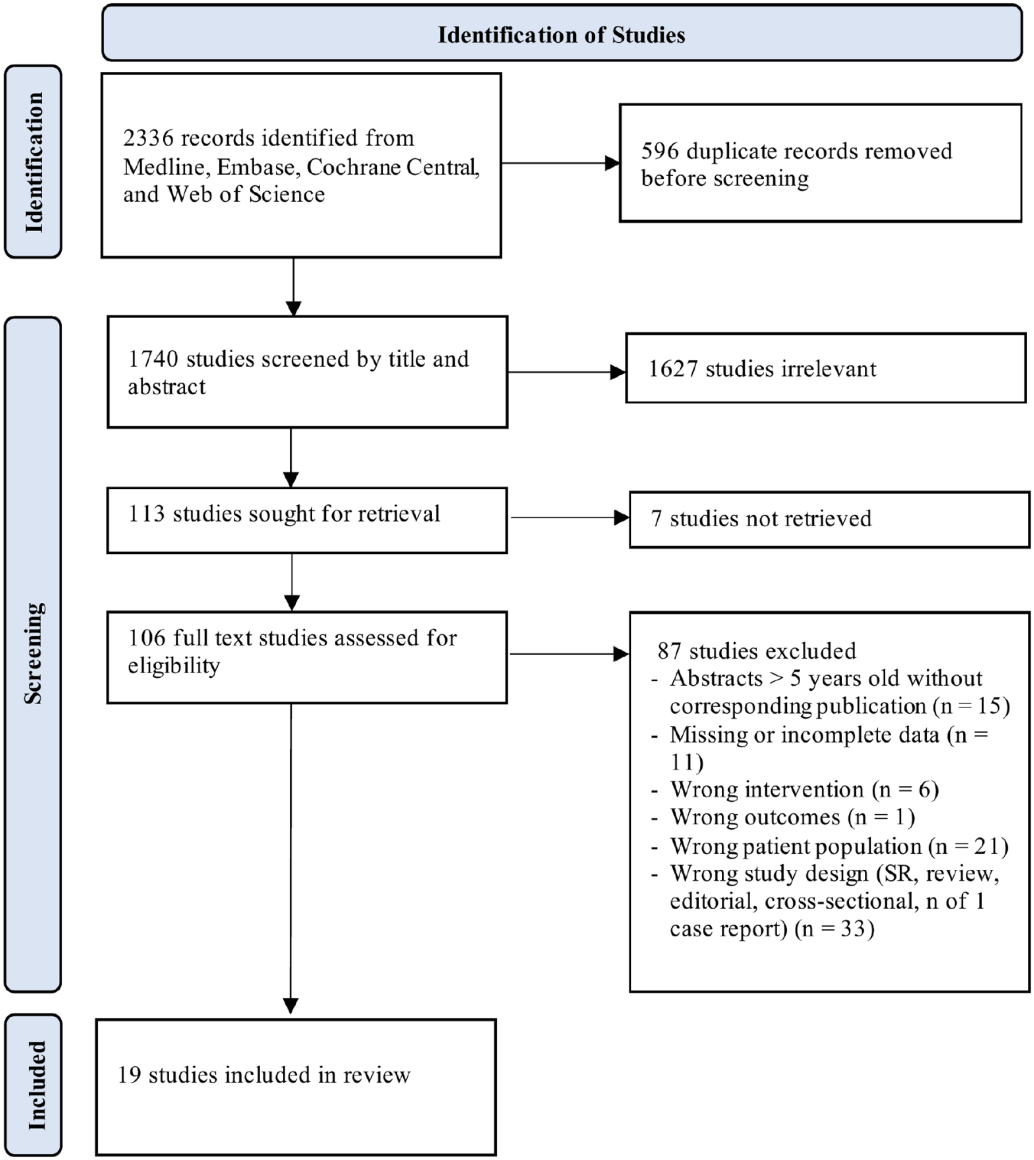
Outline of included and excluded studies. Studies excluded during full-text review with reasons are listed in Supplementary Material 2.

Continuous seizures have significant effects on the developing brain. Metabolic derangements and widespread neuronal damage begin after as little as 20 min of continuous seizures (4). As seizures continue, gamma-aminobutyric acid (GABA) inhibition declines due to pre-synaptic GABA receptor downregulation (5, 6). This decrease in GABA receptors provides a likely mechanism for why GABAergic medications, including benzodiazepines, become less effective in prolonged seizures, making them difficult to terminate (7–9).

Midazolam is a relatively inexpensive, short-acting benzodiazepine with a half-life of approximately 2 hours when administered as a bolus. Like all benzodiazepines, midazolam exerts its anticonvulsant and sedative effects by binding to the GABA_A_ receptor and increasing chloride conductance (10). However, unlike other benzodiazepines, midazolam's chemical structure and water solubility permit it to be used in a continuous infusion, and as such, it has become a mainstay in the management of RSE worldwide (10–13).

There is considerable variation in midazolam administration practices regarding starting doses, titration strategies, bolus use, and doses at which treatment is discontinued in favor of alternative third-line agents (14). Such heterogeneity in practice patterns may risk patients suffering from adverse effects of excessive midazolam administration. Alternatively, subtherapeutic doses and delayed initiation of midazolam may be ineffective in other patients and result in a prolonged duration of RSE. A key challenge in pediatric RSE is that midazolam infusions are often initiated late in the clinical course at low doses (1.0 μg/kg/min) with slow titration, likely resulting in lower efficacy from progressive impairment of GABA-mediated inhibition in prolonged seizures (8, 9, 15, 16).

To inform the future development of international protocols and clinical practice guidelines, we sought to address a key gap in the literature on the optimal midazolam prescription strategy. We conducted a systematic review of midazolam infusion strategies in pediatric RSE (ages 1 month to 21 years old), characterizing total seizure duration, midazolam infusion escalation, and the frequency of respiratory depression and hypotension. We hypothesized that the frequency of seizure termination and the incidence of hypotension and respiratory depression would be greater in studies with protocols starting at a midazolam dose of greater than 3.0 μg/kg/min or protocols incorporating more aggressive dose titration, including bolus use with infusion increases (17).

## Methods

We registered the review protocol on PROSPERO (CRD42023413038) and reported our results according to PRISMA guidelines (Supplementary Data 1) (18).

The objective of this review was to identify the optimal midazolam infusion strategy for pediatric patients with RSE. Eligible studies included randomized control trials (RCTs) and non-randomized studies published from 1990 onward involving infants and children aged 4 weeks or greater (corrected gestational age) up to 21 years, whose RSE was managed with continuous midazolam intravenous infusions (CIVs). Midazolam was infrequently used prior to 1990. Individual case studies, reviews, and editorials were excluded. Abstracts published before 2018 without an accompanying full text were also excluded. Studies including both pediatric and adult participants were included if they contained data for at least 2 pediatric participants with patient-level information.

The intervention group included all patients receiving midazolam infusions starting at 1.0–3.0 μg/kg/min, while the comparison group comprised all patients receiving midazolam infusions starting at 3.1 μg/kg/min or higher. The primary outcome was the frequency of clinical seizure termination, acknowledging that not all centers have access to continuous electroencephalogram (cEEG) monitoring. Secondary outcomes were the time to seizure cessation from midazolam CIV initiation and the frequency of respiratory depression and hypotension. When available, the severity of respiratory depression and hypotension and the interventions required were recorded.

Guided by a professional medical librarian, we searched Medline ALL (Ovid), Embase + Embase Classic (Ovid), Cochrane CENTRAL (Ovid), and Web of Science (Clarivate) using medical subject headings (MeSH), Emtree headings, and keywords (Supplementary Data 2). No studies were excluded based on language. The primary search was completed in March 2023 and updated in February 2024. Studies excluded during full-text review are listed in Supplementary Data 3.

Using Covidence, 3 authors (AA, DA, NM) independently reviewed titles and abstracts in the first stage and full-text articles in the second stage (19). A fourth author (KJ) resolved any disagreements through discussion. Pediatric data were extracted from studies including both adult and pediatric participants, provided that the study had at least 2 pediatric participants with available patient-level data (KJ, NM). All data were independently extracted by the primary authors, with disagreement resolved through discussion. All available results for each outcome measure were recorded.

We evaluated the risk of bias using a modified Cochrane Risk of Bias 2 tool (RoB2) for RCTs and the Risk Of Bias In Non-randomised Studies- of Interventions (ROBINS-I) tool for non-randomized studies (20, 21). All risk of bias assessments were performed for the primary outcome (frequency of seizure termination) independently by the primary authors (KJ, NM), with disagreements resolved through discussion. Narrative data synthesis was performed due to heterogeneity in RSE definitions, patient populations, and treatments. Descriptive statistics were performed using R Studio (R version 4.3.1) (22, 23).

## Results

### Search details

Of the initial 1,740 citations, 19 studies met the eligibility criteria (Figure 1). Most studies were non-randomized, enrolled patients with mean ages of 1.0–17.8 years, and utilized midazolam doses ranging from 1.0 to 32.0 μg/kg/min. Data from these 19 studies (3 randomized and 16 non-randomized observational trials) involving 448 pediatric patients with RSE were extracted, with representation from Europe, Asia, the Middle East, and North and Central America (Table 1) (13, 24–42).

**Table 1:** Included studies.

Author/year published/country	Study design	Total study population (*n*)	Pediatric RSE patients treated with continuous midazolam infusion (*n*)	ROB assessment
Abbaskhanian/2021/Iran (24)	Randomized control trial	70	35	Probably at high risk
Brevoord/2005/Netherlands (25)	Retrospective cohort	122	45	Critical
Daniels/2022/United States (26)	Retrospective cohort	45	45	Critical
Fallah/2007/Iran (27)	Randomized control trial	20	10	Definitely at high risk
Igartua/1999/United States (28)	Retrospective cohort	8	7	Serious
Koul/2002/Oman (29, 30)^a^	Retrospective cohort	68	38	Critical
Kumar/1992/Canada (31)	Retrospective cohort^b^	7	4	Critical
Lemerle/1995/France (32)	Retrospective cohort	4	3	Serious
Morrison/2006/United Kingdom (33)	Retrospective cohort	17	16	Serious
Omran/2009/Iran (34)	Prospective cohort	35	35	Serious
Ozdemir/2005/Turkey (35)	Prospective cohort	27	27	Critical
Patten/2015/United States (36)	Retrospective cohort	28	24	Critical
Rivera/1993/Costa Rica (13)	Prospective cohort	24	24	Serious
Singhi/2002/India (37)	Randomized control trial	40	21	Definitely at high risk
Tasker 2016/United States (38)	Prospective cohort^b^	111	42	Critical
Ulusoy/2019/Turkey (39)	Retrospective cohort	135	55	Serious
Ulvi 2002/Turkey (40)	Prospective cohort	19	5	Serious
Vasquez/2019/United States (41)	Retrospective cohort	10	7	Critical
Yamazaki/2000/Japan (42)	Retrospective cohort	10	5	Serious
Total	800		448		

^a^ Koul (2002) contains all patients included in Koul 1997. Nineteen studies met inclusion criteria, including three randomized controlled trials. Pediatric data were extracted from studies with both adult and pediatric participants. Only patients with RSE were included. If studies compared continuous midazolam infusion vs. another agent for treatment of RSE, only the data for those receiving midazolam were extracted. Risk of bias assessment was performed using a modified Cochrane Risk of Bias 2 (RoB 2) for randomized controlled trials and the Risk Of Bias In Non-randomised Studies- of Interventions (ROBINS-I) tool for non-randomized trials.

^b^ Multi-centered studies.

### Patient characteristics and RSE diagnosis

The operational definition of RSE and the medications that patients received prior to midazolam varied between studies (Table 2, Supplementary Data 4). Three studies provided a time- and medication-based RSE definition where participants exhibited seizure duration of over 60 min and failed first-line benzodiazepines and 2 second-line medications (34, 35, 39). All studies included patients with generalized convulsive seizures (GTCs), while 8 included patients with focal seizures (13, 28, 30, 34, 37, 39, 40, 42). Seizures were diagnosed clinically in 10 studies (13, 25, 27, 34–39, 42) and via continuous electroencephalogram (EEG) in 4 (26, 28, 31, 32). Study participants were predominantly infants and school-aged children, with mean ages of 1.0–17.8 years (Table 2). Two studies excluded participants with pre-existing liver or renal dysfunction, and 1 study excluded those with pre-existing chronic illness (Supplementary Data 5) (24, 27, 37). Eighteen studies included children with epilepsy (Table 2). There was significant heterogeneity in the classification of seizure etiology (Supplementary Data 5) (43). Overall, 87 children had febrile SE or central nervous system infections.

**Table 2:** Participant characteristics.

Study author	Age (years) mean (range) or mean ± SD	Female (%)	History of epilepsy (%)	Seizure type	RSE diagnosis	Seizure cessation
Abbaskhanian	3.8 ± 2.9	15 (43)	13 (37)	23 GTC, 9 NCSE, 3 myoclonic	Clinical, EEG for NCSE	Clinical
Brevoord	2.0 (0.04–16.5)^a^^,^b^^	51 (42)^b^	15 (33)	GTC	Clinical	Clinical
Daniels	<1–20^c^	16 (36)	19 (42)	NS	cEEG	cEEG
Fallah	4.2 ± 4.4	6 (60)	1 (10)	GTC	Clinical	Clinical
Igartua	4.8 ± 5.7	NS	1 (14)	3 GTC, 3 focal, 1 myoclonic	cEEG	cEEG
Koul	4.1 (0.2–14)^b^	20 (29)^b^	42 (62)^b^	43 GTC, 4 focal, 18 NCSE	Clinical, EEG for NCSE	Clinical
Kumar	12.6 (0.08–21)	3 (75)	2 (50)	3 GTC, 1 NS	Clinical ± cEEG	Clinical ± cEEG
Lemerle	1.7 (0.3–4)	0 (0)	0 (0)	2 GTC, 1 NCSE	Clinical + cEEG	Clinical + cEEG
Morrison	4.7 (0–17)^b^	NS	4 (25)	15 GTC, 2 NCSE	Clinical + EEG	Clinical + EEG
Omran	Mean 4.8 for GTC, 2.8 for focal	13 (37)	25 (71)	22 GTC, 13 focal	Clinical	Clinical
Ozdemir	5.1 ± 3.5^b^	11 (41)	10 (37)	GTC	Clinical	Clinical + EEG
Patten	7.4 (3.8–15)^b^^,^d^^	8 (33)	24 (86)^b^	NS	Clinical	Clinical
Rivera	2.2 (0.2–12)	14 (58)	14 (58)	18 GTC, 6 focal	Clinical	Clinical
Singhi	3.4 (0.2–11.5)	3 (14)	4 (19)	5 GTC, 16 focal	Clinical	Clinical + EEG
Tasker	4.5 (1.8–10.2)^b^^,^d^^	26 (48)^b^	21 (39)^b^	GTC	Clinical	Clinical or cEEG
Ulusoy	2.0 (1–4)^d^	55 (41)^b^	76 (56)^b^	150 episodes: 53 GTC, 44 generalized tonic or clonic, 49 focal	Clinical	Clinical
Ulvi	17.8 (16–20)	3 (60)	4 (80)	4 GTC, 1 focal	Clinical + EEG	Clinical + EEG
Vasquez	5.4 (0.3–16.6)^b^	8 (80)^b^	4 (40)^b^	GTC	NS	NS
Yamazaki	4.7^b^	3 (60)	4 (80)	3 GTC, 2 focal	Clinical	Clinical

NS, non-specified; GTC, generalized tonic-clonic; NCSE, non-convulsive status epilepticus.

^a^ Median, range.

^b^ Characteristics (mean unless otherwise specified) are for the overall study population. For taskers, percentages are out of a total of 54 children who received continuous anesthetic infusions.

^c^ Age categories used in the studies are as follows:<1 year old (*n* = 6), 1–6 years old (*n* = 23), 7–12 years old (*n* = 11), 13–20 years old (*n* = 5).

^d^ Median, IQR.

### Midazolam infusion titration

Children received bolus doses of midazolam (0.09–0.4 mg/kg/dose) prior to starting CIVs at 1.0–3.8 μg/kg/min (Supplementary Data 4). There was considerable variation in dose titration strategies, and only 4 studies administered boluses with each infusion increase. Three studies used rapid titration strategies, escalating CIVs every 5 min, including Morrison et al. who also used higher infusion doses, starting at 2.0 μg/kg/min and increasing by 4.0 μg/kg/min every 5 min (24, 33, 39). In Morrison et al., seizures were terminated in 14/16 (88%) of children. More often, infusions were titrated by 1.0–2.0 μg/kg/min every 10–15 min for ongoing clinical seizures.

### Seizure cessation

**Figure 2:**
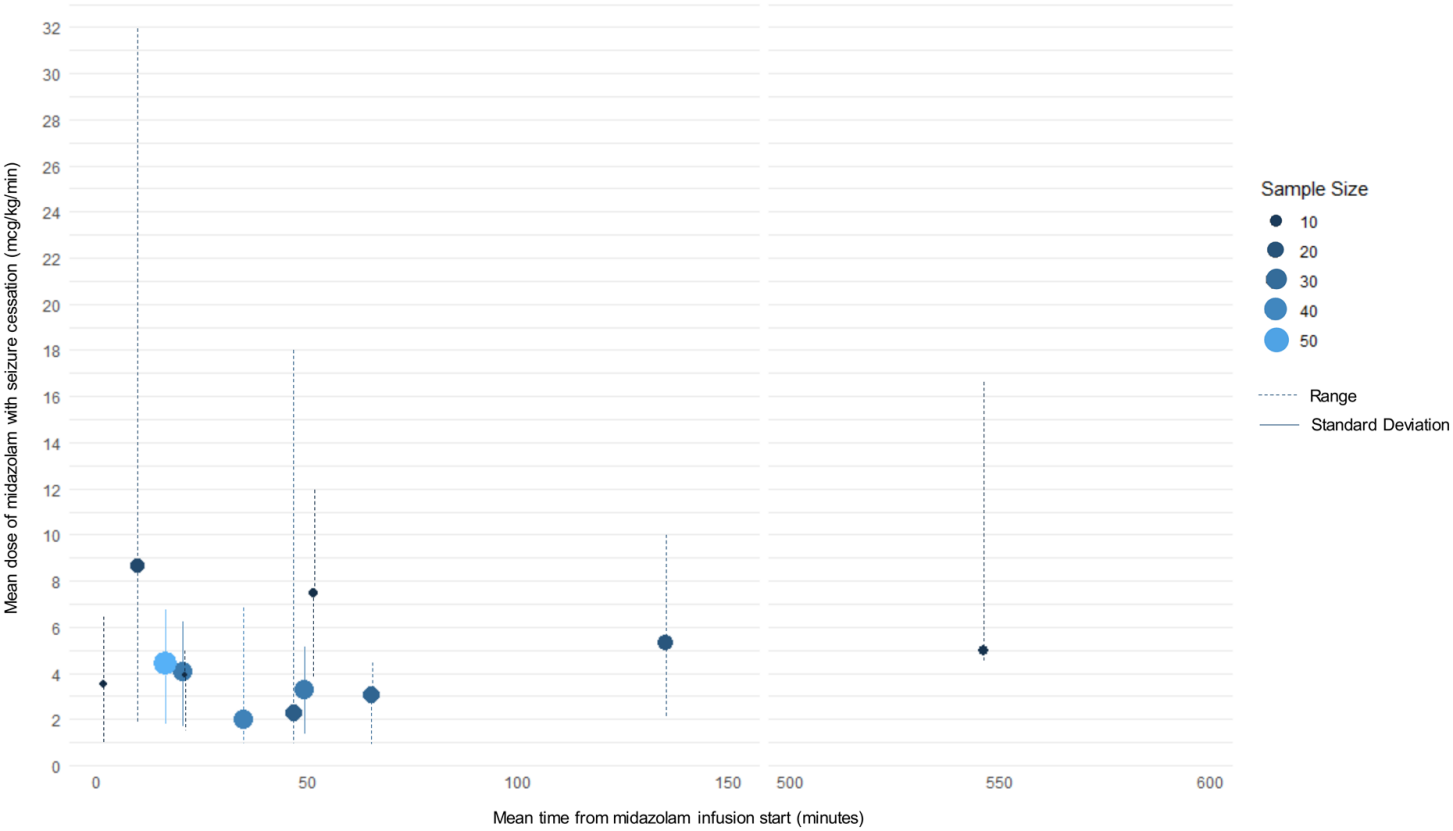
Mean dose of midazolam continuous infusion and time seizure cessation across included studies. Most studies cluster around mean midazolam continuous infusion doses of 2.0–5.0 μg/kg/min. The study patient sample size is represented by the size and color of the circles. Solid lines represent standard deviations for representative studies. Broken lines represent the range for representative studies.

Overall, midazolam CIVs aborted seizures in 363/448 (81%) children, with mean effective doses of 1.7–13.0 μg/kg/min (range 1.0–32.0 μg/kg/min) (Table 3). The mean dose administered before transitioning to another anesthetic agent varied between 1.7 and 32.0 μg/kg/min. Children who did not respond to midazolam had diverse causes of seizures, including CNS infections, neurodegenerative conditions, and metabolic disorders (Supplementary Data 5). Daniels et al. and Tasker et al. found no relationship between patient age and seizure cessation with midazolam (*n* = 87 patients) (26, 38). In 15 studies, seizures cessed in 321/399 (80%) children at mean effective doses of 2.0–5.0 μg/kg/min (13, 24–27, 29–32, 34–36, 38, 39, 41, 42). In the 12 studies that provided data, seizure cessation occurred a mean of 1.4–546 min (range 0–720 min) after CIV initiation (13, 24, 29–35, 37, 39–41). Examining the mean midazolam at seizure cessation vs. time from infusion start, effective doses for 8 studies clustered at 2.0–5.0 μg/kg/min with seizure cessation occurring between 10 and 70 min (Figure 2) (13, 24, 29–32, 34, 35, 39). In these studies, midazolam terminated seizures in 204/221 (92%) children.

**Table 3:** Midazolam infusion dosing and seizure cessation.

Study author	Seizure cessation (%)	Mean infusion dose at seizure cessation (μg/kg/min) (range)	Mean duration of infusion prior to initial seizure control (min) (range)	Max infusion dose for ineffective (μg/kg/min)
Abbaskhanian	30 (86)	4.1 ± 2.3	20.3 ± 15.6	8
Brevoord	32 (71)	4.0 (0.8–13.3)	NS	10.5 (1.7–16.7)^a^
Daniels	27 (60)	5.0 (3.3–7.9)^b^	NS	8.8
Fallah	2 (20)	2.0 (2)	NS	6.0
Igartua	6 (86)	13.0 (4–20)	78 ± 45 h^c^	24.0
Koul	37 (97)	2.0 (1–7)	34.6 (0–240)	7.0
Kumar	3 (75)	3.5 (1–6.5)	1.4	4.7
Lemerle	3 (100)	3.9 (1.7–5)	21 (1–60)	NA
Morrison	14 (88)	8.7 (2–32)	9.8 (4.8–45)	32.0
Omran	28 (80)	3.3 ± 1.9	49.2	6.0
Ozdemir	26 (96)	3.1 (1–5)	65	8.0
Patten	14 (58)	3.3 (0.83–5)^d^	NS	9.2 (4.7–15)^d^
Rivera	24 (100)	2.3 (1–18)	46.8	NA
Singhi	18 (86)	5.3 (2–10)	135 (2–720)	10.0
Tasker	30 (71)	1.7 (1–8.3)^b^	NS	3.3 (3.3–25)^b^
Ulusoy	53 (96)	3.3 (3.3–6.6)^b^	15 (9–25)^b^	15.0
Ulvi	4 (80)	7.5 (4–12)	51.3 (30–90)	21.0
Vasquez	7 (100)	5.0 (4.7–16.7)	546	NA
Yamazaki	5 (100)	2.8	(0–30)	NA

NS, not specified; NA, not applicable.

^a^ Mean, range.

^b^ Median, IQR.

^c^ Time to ultimate seizure control—patient remained in burst suppression or seizure-free and did not require further boluses or midazolam infusion increase.

^d^ Median, range.

Of the 5 studies using cEEG, only Igartua et al. reported the time to seizure control (Table 3) (26, 28, 31, 32, 38). Two studies provided data on the duration of SE prior to midazolam CIV initiation (33, 39). In Morrison et al., 15 patients experienced seizures for a mean of 354 min (range 30–1,440 min) before starting midazolam. The 2 children who did not respond to midazolam CIV had seizure durations of 180 and 1,440 min prior to midazolam. In Ulusoy et al., the median duration of SE before midazolam CIV initiation was considerably shorter at 42 min (range 30–60 min).

### Adverse events

Seventeen studies (396 children) reported respiratory and hemodynamic adverse events during midazolam CIVs (Supplementary Data 6) (13, 24, 25, 27–40, 42). In 6 studies, 47/56 (84%) children were intubated either per study protocol or before midazolam initiation (28, 31–33, 37, 40). In the remaining 11 studies, 192 children were intubated, with individual study intubation rates of 5%–72% (13, 24, 25, 27, 29, 30, 34–36, 38, 39, 42). Of the 17 studies reporting adverse events, 72 children experienced study-defined hypotension, with 23 receiving IV fluids and 49 receiving vasopressors. These rates are confounded by co-administration of phenytoin or phenobarbital and included children who experienced hypotension prior to midazolam infusion. There were 33 deaths, none of which were directly attributed to midazolam CIVs.

In the 8 studies where seizure termination occurred at doses between 2.0 and 5.0 μg/kg/min within 10–70 min (Figure 2), 42/221 (19%) children were intubated, while 18/221 (8%) experienced hypotension requiring IV fluids or no intervention (13, 24, 29–32, 34, 35, 39). These 42 intubated children include those who were intubated before midazolam CIVs and those who did not receive midazolam.

### Risk of bias

Two of the RCTs were characterized as having a definite high risk of bias (27, 37), while 1 was characterized probably at high risk of bias (Table 1, Supplementary Data 7) (24). None of the RCTs fully specified their randomization and allocation processes. Of the non-randomized studies, 8 were characterized as having a critical risk of bias (25, 26, 29–31, 35, 36, 38, 41) and 8 were characterized as having a serious risk of bias (13, 28, 32–34, 39, 40, 42). Several randomized and non-randomized studies used subjective outcomes, such as clinical determination of seizure cessation, without blinding of outcome assessors, while others had confounding effects due to the co-administration of other anti-seizure medications for sedation or seizure management (Supplementary Data 7). Notably, Abbaskhanian et al. included patients with non-convulsive status epilepticus (NCSE) but assessed seizure cessation only clinically (24).

## Discussion

We conducted a comprehensive systematic review of the literature to determine the optimal prescription strategy for pediatric RSE, aiming to better define an efficacious starting dose and titration strategy. We found 19 studies worldwide, including 3 RCTs and 16 observational trials involving 448 children with RSE treated with midazolam CIVs. Despite significant heterogeneity in starting doses and midazolam titration techniques, the mean effective dose for seizure cessation clustered between 2.0 and 5.0 μg/kg/min in 15/19 studies (Figure 2), with 80% efficacy (321/399). Midazolam-related adverse events included the requirement for mechanical ventilation in 42/221 (19%) children and hypotension requiring IV fluids or no intervention in 18/221 (8%).

Our review critically evaluates the evidence supporting midazolam infusions in pediatric RSE, with greater emphasis on dosing and bolus strategies than previous reviews. We included all studies published from 1990 onward, capturing diverse practices worldwide. While comprehensive, our review has limitations that may decrease its direct applicability to clinical practice and guideline development. Primarily, the clinical and molecular understanding of SE has changed considerably since the late 1980s to early 1990s when midazolam first entered practice (1, 2, 14, 44). Definitions of SE have evolved from including minimum seizure durations to emphasizing early aggressive seizure management (45). This means that patients with RSE in historical studies may have longer seizure durations. Importantly, variations in how RSE is defined today may limit comparison between contemporary studies (1). Across the literature, studies also use differing endpoints and access to cEEG monitoring may impact the rapidity of infusion titration and treatment durations.

Consensus guidelines for the treatment of RSE and pediatric RSE are limited by a lack of evidence. In 2020, the American Epilepsy Society Treatments Committee published a comprehensive review evaluating the efficacy of 8 anticonvulsant medications in treating refractory status epilepticus. They concluded that there is limited evidence to suggest that any one medication is more efficacious than others in seizure termination (14). Our systematic review attempts to strengthen the limited evidence for midazolam by evaluating the impact of midazolam bolus administration and the titration rate on the efficacy of seizure cessation. Unfortunately, across the 19 studies, a great deal of heterogeneity existed regarding infusion strategies and whether boluses were given with each dose titration. These variations and lack of key details limit dosing strategy comparison and identification of any subgroups that could benefit from alternative approaches. Bolus use may greatly impact the time to seizure cessation (46). Luchette et al. performed computer-based simulations of midazolam pharmacokinetics and found that infusions without boluses took longer to reach therapeutic dosing, delaying seizure cessation by 30 min (47). In addition, the use of different classification schemes for seizure etiology across studies precluded secondary subgroup analysis to determine whether certain seizure etiologies are more likely to respond to midazolam.

Beyond inter-study heterogeneity, the development of evidence-based guidelines is further limited by low certainty of evidence. All 19 studies included in this review were at probably high to critical risk of bias. Risk of bias concerns arise from subjective outcomes such as clinical determination of seizure cessation, lack of intervention blinding, and confounding effects due to the co-administration of other anti-seizure medications either for sedation or seizure management. Evaluation of clinically important respiratory adverse events is challenged by confounding factors, including concurrent or previous receipt of anti-seizure medications and studies that intubate per protocol. The incidence of midazolam-related hypotension similarly remains unclear due to confounding from phenobarbital or other anti-seizure medication administration. Limited information is provided regarding the timing of hypotension in relation to midazolam bolus and infusion escalation. Reassuringly, Morrison et al., who used a rapid midazolam escalation strategy, reported only 4 patients with hypotension (33). Two of these patients had transient hypotension associated with phenytoin and midazolam co-administration, which was corrected by 10 ml/kg of IV fluids, while 2 were already receiving vasoactive medication prior to midazolam titration without any increase in their dosing during midazolam titration. Finally, we note the potential bias and limitations of including case series as observational trial data.

With the potential decrease in efficacy of midazolam in prolonged seizures from GABAergic downregulation, it is tempting to abandon midazolam in favor of alternative agents. However, the lack of standardized protocols, questionable use of boluses, and variable data reporting limit conclusions. Despite the above limitations, our review suggests a therapeutic window for midazolam CIVs between 2.0 and 5.0 μg/kg/min and indicates that starting the infusion within this therapeutic window may achieve earlier seizure cessation. Given that midazolam is a relatively inexpensive and widely available medication with potentially fewer respiratory and hemodynamic consequences than phenobarbital or propofol, we advocate for further research to address important dosing and efficacy questions (38, 48). Midazolam may be more effective when used earlier and rapidly titrated with boluses. Our systematic review highlights the vast differences in approaches to continuous midazolam infusions in treating RSE but also differences in how SE and RSE are defined and how seizure etiology is classified. Without consensus on these definitions and standardization of boluses within protocols, comparison across studies and collaboration across countries will remain challenging. Research collaboratives, such as the Pediatric Status Epilepticus Research Group, will play a key role in achieving this standardization (1). Research investigating midazolam and other third-line anesthetics will also need to consider the timing of initiating these medications and clinician hesitation to use anesthetics, including at higher doses, for fear of respiratory depression, hypotension, or potential increased risk of mortality (49).

## Conclusions

Midazolam is a commonly used anesthetic infusion for the treatment of pediatric RSE, yet there is limited evidence guiding treatment protocols and clinical practice. Data from 19 studies involving 448 children illustrate significant heterogeneity in infusion dosing, bolus use, seizure termination effectiveness, and incidence of respiratory and hemodynamic adverse events. Our findings suggest a potential therapeutic window at rates of 2.0–5.0 μg/kg/min (0.12–0.30 mg/kg/h), with limited adverse risks. Earlier seizure cessation may occur by targeting this therapeutic window by initiating treatment at higher doses than the typically used 1.0 μg/kg/min (0.06 mg/kg/h) or by rapidly escalating the dose.

## Data Availability

The original contributions presented in the study are included in the article/Supplementary Material; further inquiries can be directed to the corresponding author.
