# Supplementary material for: Midazolam infusions for therapeutic management of pediatric refractory status epilepticus: a systematic review
Source: Front Pediatr. 2025 Apr 14;13:1507325. doi: 10.3389/fped.2025.1507325 (PMC12034653; doi:10.3389/fped.2025.1507325)
Supplement: Supplementary file 1 [file Datasheet1.pdf]

Supplementary Material

1. Search Strategy
2. Studies Excluded
3. Midazolam Infusion Protocols
4. Seizure Etiologies
5. Midazolam Infusion Complications
6. Risk of Bias for Included Studies
7. Refractory Status Epilepticus Clinical Practice Guidelines and Literature Reviews

Supplementary Material 1: Search Strategy and Information Sources

Search Summary

**Database [Platform]** *Searches run March 13, 2023*	**Results**
MEDLINE(R) ALL [OVID] <1946 to March 10, 2023>	343
Embase Classic+Embase [OVID] 1947 to 2023 week 09	1377
EBM Reviews - Cochrane Central Register of Controlled Trials February 2023	62
Web of Science Core Collection – All databases March 13, 2023	413
**TOTAL**	**2195**
**TOTAL with 587 duplicates removed in Covidence**	**1608**

The search strategy was developed by a professional medical librarian trained in knowledge synthesis. The search was executed in the following bibliographic databases on March 13, 2023: MEDLINE(R) ALL [OVID] (1946 to March 10, 2022); Embase+Embase Classic (1974–2023 week 10); Cochrane Central Register of Controlled Trials (February 2023) via Ovid; and Web of Science Core Collection All Databases (March 13, 2023) via Clarivate. The search strategy consisted of both controlled vocabulary, such as the National Library of Medicine's MeSH (Medical Subject Headings) and Emtree Subject Headings (Embase), and keywords. Date limits of 1990-2023 were applied. No language limits or other study type filters were applied.

Search Results

Ovid MEDLINE(R) ALL <1946 to March 10, 2023>

#	Searches
**1**	Status Epilepticus/
**2**	("refractory status epilepticus" or RSE or "persistent seiz*" or "persistent status epilepticus" or "New-onset refractory status epilepticus" or NORSE or " De novo cryptogenic refractory multifocal febrile status epilepticus" or "convulsive refractory status epilepticus" or CRSE or "refractory repetitive partial seizure*" or "Prolonged seizure*" or "Epilepsia partialis continua" or "Status epilep*" or "Seizure termination" or "febrile seizure*" or "refractory convulsive status epilepticus" or RCSE).tw,kf.
**3**	1 or 2
**4**	exp Midazolam/ or (Midazolam* or Dormicum or Versed or "Ro 21-3981" or "Ro 213981" or "high-dose midazolam" or "low-dose midazolam").tw,kf.
**5**	3 and 4
**6**	exp adolescent/ or exp child/ or infant/ or *pediatrics/ or (adolescen* or babies or baby or boy? or boyhood or girlhood or child* or girl? or infan* or juvenil* or kid? or minors or minors* or paediatric* or pediatric* or preschool* or puber* or pubescen* or school* or teen* or toddler? or underage? or under-age? or youth*).tw,kf.

**7**	"Intensive Care Units, Pediatric"/ or (p$ediatric* adj3 ("intensive care" or "ICU" or "PICU" or "CICU" or "critical care" or "acute care")).tw,kf.
**8**	6 or 7
**9**	5 and 8
**10**	limit 9 to yr="1990 - 2023"
**11**	remove duplicates from 10

Embase Classic+Embase <1947 to 2023 Week 10>

**#**	**Searches**
**1**	*Epileptic state/
**2**	("refractory status epilepticus" or RSE or "persistent seiz*" or "persistent status epilepticus" or "New-onset refractory status epilepticus" or NORSE or " De novo cryptogenic refractory multifocal febrile status epilepticus" or "convulsive refractory status epilepticus" or CRSE or "refractory repetitive partial seizure*" or "Prolonged seizure*" or "Epilepsia partialis continua" or "Status epilep*" or "Seizure termination" or "febrile seizure*" or "refractory convulsive status epilepticus" or RCSE).tw,kf.
**3**	1 or 2
**4**	exp Midazolam/ or (Midazolam* or Dormicum or Versed or "Ro 21-3981" or "Ro 213981" or "high-dose midazolam" or "low-dose midazolam").tw,kf.
**5**	3 and 4
**6**	exp adolescent/ or exp child/ or infant/ or *pediatrics/ or (adolescen* or babies or baby or boy? or boyhood or girlhood or child* or girl? or infan* or juvenil* or kid? or minors or minors* or paediatric* or pediatric* or preschool* or puber* or pubescen* or school* or teen* or toddler? or underage? or under-age? or youth*).tw,kf.
**7**	"pediatric intensive care unit"/ or (p$ediatric* adj3 ("intensive care" or "ICU" or "PICU" or "CICU" or "critical care" or "acute care")).tw,kf.
**8**	6 or 7
**9**	5 and 8
**10**	limit 9 to yr="1990 - 2023"
**11**	remove duplicates from 10

EBM Reviews - Cochrane Central Register of Controlled Trials February 2023

#	Searches
1	exp Status Epilepticus/
2	("refractory status epilepticus" or RSE or "persistent seiz*" or "persistent status epilepticus" or "New-onset refractory status epilepticus" or NORSE or " De novo cryptogenic refractory multifocal febrile status epilepticus" or "convulsive refractory status epilepticus" or CRSE or "refractory repetitive partial seizure*" or "Prolonged seizure*" or "Epilepsia partialis continua" or "Status epilep*" or "Seizure termination" or "febrile seizure*" or "refractory convulsive status epilepticus" or RCSE).tw,kw.
3	1 or 2
4	exp Midazolam/ or (Midazolam* or Dormicum or Versed or "Ro 21-3981" or "Ro 213981" or "high-dose midazolam" or "low-dose midazolam").tw,kw.
5	3 and 4
6	exp adolescent/ or exp child/ or infant/ or *pediatrics/ or (adolescen* or babies or baby or boy? or boyhood or girlhood or child* or girl? or infan* or juvenil* or kid? or minors or minors* or paediatric* or pediatric* or preschool* or puber* or pubescen* or school* or teen* or toddler? or underage? or under-age? or youth*).tw,kw.
7	exp "Intensive Care Units, Pediatric"/ or (p$ediatric* adj3 ("intensive care" or "ICU" or "PICU" or "CICU" or "critical care" or "acute care")).tw,kw.
8	6 or 7
9	5 and 8
10	limit 9 to yr="1990 - 2023"
11	remove duplicates from 10

Web of Science Core Collection – All databases March 13, 2023

1	TS=("refractory status epilepticus" or RSE or "persistent seiz*" or "persistent status epilepticus" or "New-onset refractory status epilepticus" or NORSE or " De novo cryptogenic refractory multifocal febrile status epilepticus" or "convulsive refractory status epilepticus" or CRSE or "refractory repetitive partial seizure*" or "Prolonged seizure*" or "Epilepsia partialis continua" or "Status epilep*" or "Seizure termination" or "febrile seizure*" or "refractory convulsive status epilepticus" or RCSE)
2	TS=(Midazolam* or Dormicum or Versed or "Ro 21-3981" or "Ro 213981" or "high-dose midazolam" or "low-dose midazolam")
3	#2 AND #1
4	TS=(adolescen* or babies or baby or boy? or boyhood or girlhood or child* or girl? or infan* or juvenil* or kid? or minors or minors* or paediatric* or pediatric* or preschool* or puber* or pubescen* or school* or teen* or toddler? or underage? or under-age? or youth*)

**5**	TS=((p$ediatric* NEAR/3 "intensive care" or "ICU" or "PICU" or "CICU" or "critical care" or "acute care"))
**6**	#5 OR #4
**7**	#6 AND #3
**8**	#6 AND #3 and Article (Document Types)

Search Update: February 2024

The above search was repeated on February 1, 2024, with limits changed to 2024.

**Database [Platform]** *Searches run February 1, 2024*	**Results**
MEDLINE(R) ALL [OVID] <March 10, 2023 to February 1, 2024>	14
Embase Classic+Embase [OVID] 2023 week 09 to 2024 week 05	103
EBM Reviews - Cochrane Central Register of Controlled Trials January 2024	4
Web of Science Core Collection – All databases February 1, 2024	20
**TOTAL**	**141**
**TOTAL with 23 duplicates removed in Covidence**	**118**

Supplementary Material 2: Studies Excluded at Full Text Review

Abstracts over 5 years old were excluded if the data were not subsequently published as a full text article.

	Author	Year	Reason Excluded	Notes
1	Fernandez	2014	Abstract > 5 yrs old	
2	Goodkin	2014	Abstract > 5 yrs old	
3	Kravljanac	2013	Abstract > 5 yrs old	
4	Kravljanac	2009	Abstract > 5 yrs old	Also: missing or incomplete data
5	Kravljanac	2014	Abstract > 5 yrs old	
6	Kravljanac	2016	Abstract > 5 yrs old	Also: wrong patient population
7	Kwon	2009	Abstract > 5 yrs old	Also: cross-sectional study
8	Langer	2012	Abstract > 5 yrs old	
9	Loddenkemper	2014	Abstract > 5 yrs old	
10	Loddenkemper	2015	Abstract > 5 yrs old	
11	Muro	2012	Abstract > 5 yrs old	
12	Olgac Dundar	2015	Abstract > 5 yrs old	Also: missing or incomplete data
13	Shah	2013	Abstract > 5 yrs old	Also: wrong patient population (only SRSE)
14	Tasker	2015	Abstract > 5 yrs old	Also: missing or incomplete data
15	Fayyazi	2012	Abstract > 5 yrs old	
16	Tu	2013	Full text not available	
17	Agadi	2011	Full text not available	
18	Galdames Poblete	1994	Full text not available	
19	Sakaue	2005	Full text not available	
20	Singhi	2007	Full text not available	
21	Vittorini	2008	Full text not available	
22	Yoshikawa	2004	Full text not available	
23	Hayashi	2007	Missing or incomplete data	
24	Kravljanac	2015	Missing or incomplete data	
25	Lohr	2000	Missing or incomplete data	
26	Mansoor	2022	Missing or incomplete data	
27	Nobutoki	2005	Missing or incomplete data	
28	Saz	2011	Missing or incomplete data	
29	Mahvelati	2007	Missing or incomplete data	
30	Cavusoglu	2021	Missing or incomplete data	
31	Reza Salehiomran	2011	Missing or incomplete data	
32	Sajda	2021	Missing or incomplete data	Abstract only, unclear how they determined seizure cessation
33	Fayyazi	2011	Missing or incomplete data	
34	Minagawa	1998	Wrong intervention	Received midazolam infusion as 2nd line if previously had a good response to midazolam as 1st line
35	Gregory	2022	Wrong intervention	Studied high dose vs low dose midazolam bolus and proportion of patients requiring intubation

36	Kincaid	2017	Wrong intervention	Studied a pre-refractory SE treatment protocol, looked at success of protocol implementation
37	Lampin	2010	Wrong intervention	Intubation induction done with midazolam
38	Savary	2022	Wrong intervention	Looked at patients receiving multiple 2nd line medications and whether they were more or less likely to need midazolam infusions than those receiving only one 2nd line medication
39	Mahmoudian	2005	Wrong outcome	All patients received same dose of midazolam infusion, looked at seizure cessation after 20 min only
40	Fayyazi	2012	Wrong patient population	Not RSE
41	Ibrahim	2003	Wrong patient population	Not RSE
42	Nishiyama	2018	Wrong patient population	Not RSE
43	Parent	1994	Wrong patient population	Only 1 pediatric patient
44	Singh	2018	Wrong patient population	Adult patients
45	Yoshikawa	2000	Wrong patient population	Not RSE, midazolam infusion given first line
46	Chiu	2022	Wrong patient population	Adult patients
47	Kravljanac	2010	Wrong patient population	Not RSE
48	Masapu	2019	Wrong patient population	Adult patients
49	Hamano	2019	Wrong patient population	Not RSE
50	Hamano	2003	Wrong patient population	Not RSE, missing or incomplete data
51	Kang	2015	Wrong patient population	Adult patients, 3 patients under 21 yo but missing or incomplete data
52	Kazmi	2021	Wrong patient population	Not RSE
53	Kumar	1992	Wrong patient population	Not RSE
54	Prasad	2001	Wrong patient population	Only 1 pediatric patient
55	Vasquez	2021	Wrong patient population	SRSE patients only, full text publication of study 2022
56	Bellante	2016	Wrong patient population	Adult patients
57	Ito	2017	Wrong patient population	Not RSE
58	Vasquez	2018	Wrong patient population	SRSE patients only, platform presentation of study 307
59	Minagawa	2003	Wrong patient population	Not RSE
60	Babl	2009	Wrong study design	Cross-sectional
61	Chen	2018	Wrong study design	Cross-sectional
62	Federman	2009	Wrong study design	N of 1 case report
63	Gilbert	1999	Wrong study design	Review
64	Hanley	1998	Wrong study design	Review
65	Holmes	1999	Wrong study design	Review
66	Hussain	2007	Wrong study design	Cross-sectional
67	Iwasaki	2010	Wrong study design	Pharmacology study
68	Koul	2000	Wrong study design	Cross-sectional
69	Meyer	2023	Wrong study design	Cross-sectional
70	Morrison	2008	Wrong study design	Editorial

71	Peariso	2022	Wrong study design	Editorial
72	Pellock	1998	Wrong study design	Review
73	Sahin	2021	Wrong study design	Cross-sectional
74	Tasker	2006	Wrong study design	Editorial
75	Tully	2015	Wrong study design	Cross-sectional
76	Wilkes	2012	Wrong study design	Review
77	Wilkes	2014	Wrong study design	Review
78	Cardoso	2013	Wrong study design	Cross-sectional
79	Acvedo	2011	Wrong study design	Cross-sectional, also missing or incomplete data
80	Chaves-Carballo	2002	Wrong study design	No intervention
81	Jayachandran	2012	Wrong study design	Cross-sectional
82	Jelinek	1994	Wrong study design	Editorial
83	Luchette	2019	Wrong study design	Pharmacology study
84	Medjo	2009	Wrong study design	Cross-sectional
85	Minagawa	1995	Wrong study design	N of 1 case report
86	Minagawa	2005	Wrong study design	Review
87	Tully	2013	Wrong study design	Cross-sectional
88	Abbaskhanian	2016	Wrong study design	Clinical study protocol registry only
89	Wilmshurst	2018	Wrong study design	Clinical study registry only, also wrong patient population

Studies excluded at secondary full text review (February 2024)

	Author	Year	Reason Excluded	Notes
90	Srivastava	2023	Wrong intervention	Investigated compliance with institutional status epilepticus treatment algorithm
91	Soydan	2023	Wrong patient population	SRSE patients only, no doses of midazolam given
92	Morais	2023	Wrong study design	Cross sectional study describing status epilepticus prevalence and patient characteristics, no doses of midazolam given
93	Sadek	2023	Wrong study design	Clinical trial registry only
94	Vossler	2023	Wrong study design	Editorial

RSE: refractory status epilepticus, SRSE: super-refractory status epilepticus

Supplementary Material 3: Midazolam Titration and Bolus Protocols

Study Author	Treatment before midazolam CI	Bolus prior to infusion start (mg/kg) (Range)	Infusion starting dose (mcg/kg/min)	Dose titration (mcg/kg/min), interval of titration (min)	Bolus with rate up-titrations (mg/kg)
Abbaskhanian[a]	DZP + PHY or PHB	0.2 (max 10 mg)	1	1, (5)	NS
Brevoord	DZP and/or MDZ + PHY	0.2	1.7	1.7, (10)	0.1
Daniels[a]	LZP + PHY	0.1-0.2	1.7	0.8, (15)	NS
Fallah	DZP + PHY + PHB	0.15	1	1, (15)	NS
Igartua	BZP + PHY + PHB	0.15	1-2	1-2, (15)	0.15, by discretion
Koul	DZP + PHY and/or PHB, 2 received VPA	0.15	1	NS	NS
Kumar	DZP and/or LZP + PHY and/or PHB	0.20, (0.02-0.42)[b]	NS	NS	NS
Lemerle	DZP or clonazepam + PHY + PHB	0.3-0.4	NS	NS	NS
Morrison	2 or more AEDs (most often BZP + PHY)	0.5	2	4, (5)	0.5 for first increase, 0.1 thereafter
Omran	DZP + PHY + PHB	0.15	1	1, (15)	NS
Ozdemir	DZP + PHY + PHB	0.2	1	1, (15)	NS
Patten[a]	LZP + DZP + PHY + PHB	NS	NS	NS	NS
Rivera	DZP + PHY + PHB	0.15	1	NS, (15)	NS
Singhi	DZP + PHY + another BZP	0.2	2	NS	NS
Tasker[a]	Median 4 AEDs (most 2 BZP + 2 non-BZP)	0.11, (0.09-0.22)[c]	1.7 (1-3.8)[b]	NS	NS
Ulusoy[a]	MDZ + PHY or LEV, +/- patient's home ASM	0.2	1.7-3.3	1.7, (5)	0.1-0.2
Ulvi	DZP + PHY + PHB	0.2	1	1, (15)	NS
Vasquez[a]	NS	0.1, (0.09-0.13)[b]	1.3-2.5	NS	NS
Yamazaki	DZP + PHY	0.1-0.15	1-2	NS	NS

CI: continuous infusion, NS: non-specified, RSE: refractory status epilepticus, CSE: convulsive status epilepticus, NCSE: non-convulsive status epilepticus, BZP: benzodiazepine, DZP: diazepam, PHY: phenytoin, PHB: phenobarbital, MDZ: midazolam, LEV: levetiracetam, LZP: lorazepam, ASM: anti-seizure medication

[a] Study conducted after 2012 when the definition of status epilepticus changed to seizure duration greater than 5 minutes.

[b] Mean, Range, [c] Median, IQR

Supplementary Material 4: Seizure Etiologies of Patients Receiving Midazolam Continuous Infusions

Study Author	Excluded seizure etiologies and patient conditions	Included seizure etiologies of all patients receiving midazolam infusions, n (%)	Etiologies of patients that did not respond to midazolam, n (%)
Abbaskhanian	Liver disease, coagulopathy, metabolic disease, or traumatic seizures. Known hypotension, or AV block.	Febrile (excluding CNS infections): 3 (9)<br>Acute symptomatic: 7 (20)<br>Remote symptomatic: 12 (35)<br>Idiopathic/ cryptogenic: 7 (20)<br>Acute on remote: 6 (17)	NS
Brevoord	83 of 205 patients admitted for generalized status epilepticus were excluded from the overall study because of protocol violations or missing treatment data.	Febrile: 18 (40)<br>Acute symptomatic: 12 (27)<br>Idiopathic: 14 (31)<br>Neurodegenerative: 1 (2)	Febrile: 2 (15)<br>Acute symptomatic: 7 (54)<br>Idiopathic: 3 (23)<br>Neurodegenerative: 1 (8)
Daniels	12 of 57 patients receiving midazolam infusion excluded for incomplete records, admitted to NICU at start of treatment, or history of treatment failure with midazolam.	History of epilepsy: 19 (42)<br>Stroke/ trauma/ cardiac arrest: 7 (16)<br>Febrile: 6 (13)<br>Autoimmune encephalitis: 5 (11)<br>Toxic-metabolic: 4 (9)<br>CNS infection: 2 (4)<br>CNS neoplasm: 2 (4)	NS
Fallah	Liver or renal dysfunction or 2nd admission with status epilepticus. Hypotension, cardiac arrythmias or AV block.	Symptomatic epilepsy: 9 (90)<br>Idiopathic epilepsy: 1 (10)	Symptomatic epilepsy: 6 (75)<br>Idiopathic epilepsy: 2 (25)
Igartua	NA	Encephalitis: 2 (29)<br>Idiopathic: 1 (14)<br>Post-open-heart bypass: 1 (14)<br>Pyruvate dehydrogenase deficiency: 1 (14)<br>Mitochondrial disorder: 1 (14)<br>Neonatal seizures: 1 (14)	Mitochondrial disorder: 1 (100)
Koul *	NA	Meningitis or encephalitis: 14 (21)<br>Idiopathic: 30 (44)<br>Febrile: 6 (9)<br>Neurodegenerative: 5 (7)<br>Cerebral dysgenesis: 5 (7)<br>Neurocutaneous disorder: 3 (4)<br>Other: 5 (7)	Batten Disease: 1 (100)
Kumar	NA	Theophylline overdose: 1 (25)<br>Encephalitis: 1 (25)<br>Neurodegenerative: 1 (25)<br>Febrile: 1 (25)	Encephalitis: 1 (100)
Lemerle	NA	Post cardiac arrest: 1 (33)<br>Leukemia with recent intrathecal chemotherapy: 1 (33)	All seizures stopped.

		Chlamydia associated vasculitis: 1 (33)	
Morrison	NA	Meningitis or encephalitis: 4 (25)<br>Structural brain lesion: 4 (25)<br>Subdural hematoma: 2 (13)<br>Post cardiac arrest: 2 (13)<br>Idiopathic: 4 (25)	Subdural hematoma: 1 (50)<br>Post cardiac arrest: 1 (50)
Omran	Need for urgent neurosurgical intervention, electrolyte disturbances. Any conditions causing hypotension or respiratory insufficiency.	NS	NS
Ozdemir	NA	Meningitis or encephalitis: 12 (44)<br>Drug overdose: 1 (4)<br>Subarachnoid hemorrhage: 1 (4)<br>Hypoxic injury with known epilepsy: 4 (15)<br>Brain tumor: 1 (4)<br>Idiopathic: 6 (22)<br>Neurodegenerative: 2 (8)	Meningoencephalitis: 1 (100)
Patten	Patients transferred from another hospital intubated and receiving benzodiazepines were excluded.	NS	NS
Rivera	NA	Pre-existing epilepsy: 14 (58)<br>Meningitis or encephalitis: 3 (13)<br>Brain abscess: 1 (4)<br>Idiopathic: 6 (25)	All seizures stopped.
Singhi	Excluded neonates and children with chronic illnesses including existing cardiac or respiratory disease.	Meningoencephalitis: 10 (48)<br>Late hemorrhagic disease of newborn: 4 (19)<br>Hepatic encephalopathy: 1 (5)<br>Other: 6 (29)	NS
Tasker	NA	Symptomatic: 30 (56)<br>Genetic/ metabolic: 10 (19)<br>Idiopathic/ other: 14 (26)	NS
Ulusoy	NA	NS	NS
Ulvi	NA	Idiopathic/ unknown: 5 (100)	Idiopathic: 1 (100)
Vasquez	NA	Idiopathic/ unknown: 6 (60)<br>Structural brain lesion: 2 (20)<br>Metabolic disease: 1 (10)<br>Genetic: 1 (10)	NS
Yamazaki	NA	Idiopathic: 4 (80)<br>Febrile: 1 (20)	All seizures stopped.

CNS: central nervous system, NS: not specified, NA: not applicable (no etiologies excluded)

[*] For overall cohort of all patients with status epilepticus including those that did not receive midazolam infusion.

[ǂ] For n = 54 patients that received continuous anesthetic infusions.

Supplementary Material 5: Complications of patients receiving midazolam continuous infusions

Study Author	Intubation (%)	Hypotension (%)	Intervention for hypotension	All cause mortality (%)
Abbaskhanian	8 (23)	7 (20)	NS	NS
Brevoord	52 (43) [ǂ]	0	NA	7 (6) [ǂ]
Daniels	NS	8 (18)	Vasoactives	NS
Fallah	7 (70)	0	NA	NS
Igartua	7 (100)[*]	0	NA	1 (13)
Koul	2 (5)	0	NA	1 (3)
Kumar	4 (100)[*]	0	NA	0
Lemerle	3 (100)[*]	0	NA	0
Morrison	15 (88)[*]	4 (25)	IV fluids, vasoactives	2 (12)
Omran	3 (9)	8 (23)	IV fluids	0
Ozdemir	0	0	NA	5 (19)
Patten	17 (61) [†]	17 (61)	Vasoactives	1 (4)
Rivera	0	0	NA	0
Singhi	13 (62) [*]	8 (38)	IV Fluids, vasoactives	8 (38)
Tasker	80 (72) [ǂ]	12 (29)[§]	Vasoactives	4 (4) [ǂ]
Ulusoy	22 (16) [ǂ]	3 (2) [ǂ]	IV Fluids	3 (2)
Ulvi	5 (100) [*]	0	NA	1 (20)
Vasquez	NS	NS	NS	NS
Yamazaki	1 (20)	0	NA	0

NS: not specified, NA: not applicable

[*]Includes intubations occurring before start of midazolam infusion.

[†]Overall incidence of intubation and hypotension for patients receiving midazolam infusion or midazolam infusion and pentobarbital (those that failed midazolam therapy). The study does not specify if hypotension and respiratory depression occurred with midazolam or only after pentobarbital was administered. Of patients who only received midazolam, 41% were intubated and 41% had hypotension.

[ǂ] For the overall population, including those who did not receive continuous midazolam.

[§]Includes patients who failed midazolam and received pentobarbital.

Supplementary Material 6: Risk of Bias for Included Studies

A

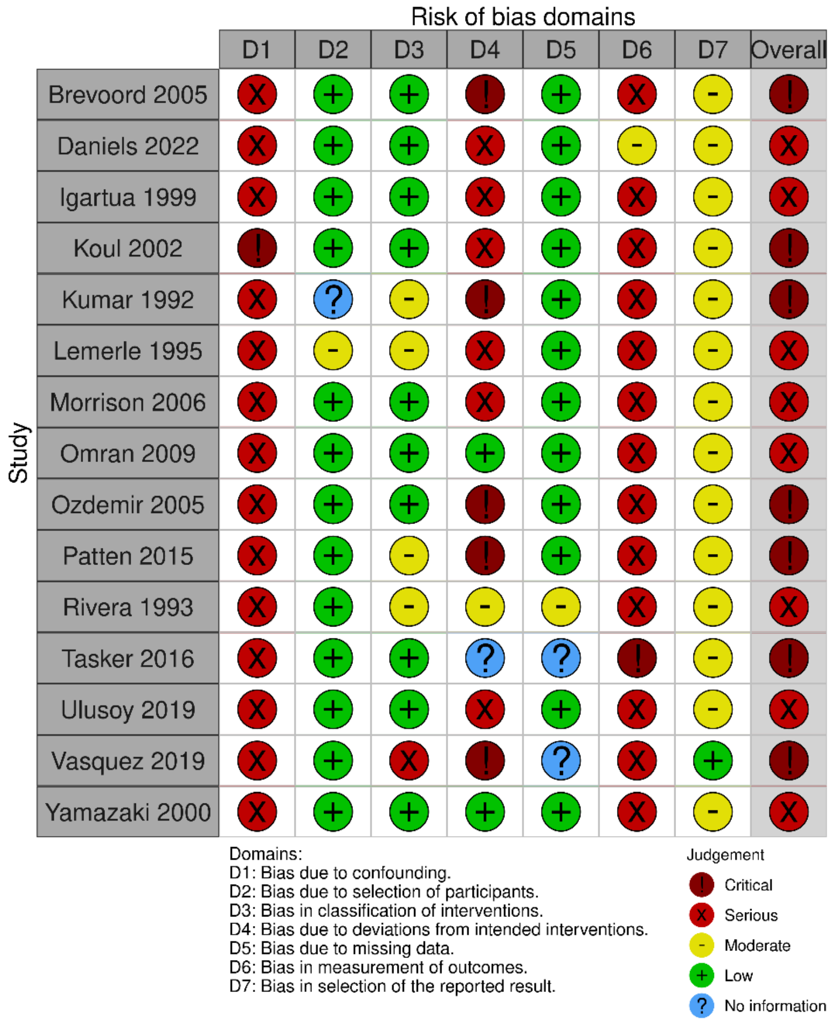

B

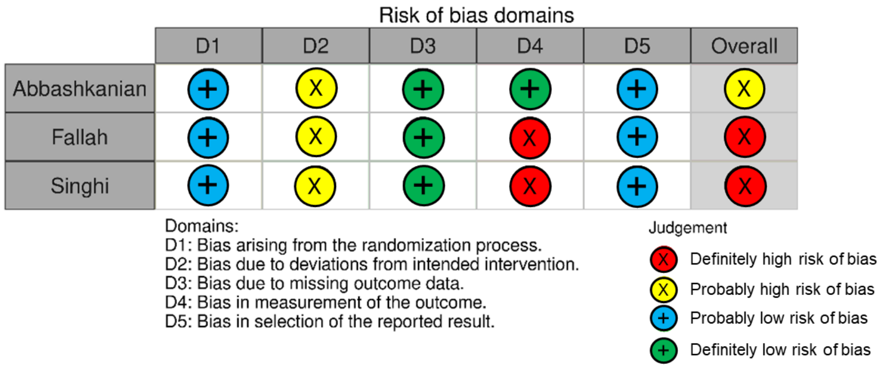

Risk of bias assessment for non-randomized trials (A) using Cochrane ROBINS-I and for randomized trials (B) using a modified RoB-2. All risk of bias assessments were done for the primary outcome, frequency of seizure cessation.

Supplementary Material 7: Refractory Status Epilepticus Clinical Practice Guidelines and Literature Reviews

Reference	Definition of RSE	Treatment Algorithm and Dosages
Neurocritical Care Society, Guidelines for the Evaluation and Management of Status Epilepticus (2012) (1)	Ongoing clinical or electrographic seizures despite adequate dose of initial BZP followed by 2nd acceptable AED. Most experts do not consider seizure duration as a criteria for RSE.	Recommend seizure control within 60 min of seizure onset.<br>Divided into 1st, 2nd, 3rd, and 4th line therapy.<br>RSE therapy is 3rd and 4th line.<br><br>Either repeat bolus AED or start CI. Start CI if attempted repeat bolus AED is not successful.<br><br>Not enough data to recommend a preferred CI agent. Options include midazolam, propofol, pentobarbital, or thiopental. Treatment intensity is usually guided by cEEG.<br><br>Midazolam Recommendations<br>• Bolus 0.2 mg/kg at rate of 2 mg/min<br>• Then CI of 0.05-2 mg/kg/hr<br>• If breakthrough SE: give 0.1-0.2 mg/kg bolus, inc CI rate by 0.05-0.1 mg/kg/hr q3-4 hr
American Epilepsy Society, Evidence-Based Guideline: Treatment of Convulsive Statue Epilepticus in Children and Adults (2016) (2)	Seizures are refractory when seizure duration reaches 40-60 minutes (3rd therapy phase)<br><br>Treatment of RSE is beyond the guideline scope.	No clear evidence to guide therapy in this phase.<br><br>Clinicians may choose to repeat 2nd line therapy or start general anesthesia with thiopental, midazolam, pentobarbital or propofol. All anesthetics should be titrated with cEEG.<br><br>No specific recommendations for treatment doses.
American Epilepsy Society, Treatment of Refractory Convulsive Status Epilepticus: A Comprehensive Review (2020) (3)	Failure of 2 adequately dosed AEDs in different drug classes (e.g., BZP, PHY).	Insufficient evidence to guide treatment recommendations. Literature review discusses various 2nd and 3rd line agents including levetiracetam, valproate, ketamine, midazolam, phenobarbital, propofol.<br><br>No specific recommendations for treatment escalation strategy. Most

		common midazolam doses are provided.<br>• Loading dose 0.2-0.5 mg/kg IV<br>• CI: 0.1-2 mg/kg/hr or 2-40 mcg/kg/min

BZP: benzodiazepine, AED: anti-epileptic drug, RSE: refractory status epilepticus, CI: continuous infusion. cEEG: continuous electroencephalogram, PHY: phenytoin

References

1. Brophy GM, Bell R, Claassen J, Alldredge B, Bleck TP, Glauser T, LaRoche SM, Riviello JJ, Shutter L, Sperling MR, et al. Guidelines for the Evaluation and Management of Status Epilepticus. *Neurocritical Care* (2012) 17:3–23. doi: 10.1007/s12028-012-9695-z

2. Glauser T, Shinnar S, Gloss D, Alldredge B, Arya R, Bainbridge J, Bare M, Bleck T, Dodson WE, Garrity L, et al. Evidence-Based Guideline: Treatment of Convulsive Status Epilepticus in Children and Adults: Report of the Guideline Committee of the American Epilepsy Society. *Epilepsy Curr* (2016) 16:48–61. doi: 10.5698/1535-7597-16.1.48

3. Vossler DG, Bainbridge JL, Boggs JG, Novotny EJ, Loddenkemper T, Faught E, Amengual-Gual M, Fischer SN, Gloss DS, Olson DM, et al. Treatment of Refractory Convulsive Status Epilepticus: A Comprehensive Review by the American Epilepsy Society Treatments Committee. *Epilepsy Curr* (2020) 20:245–264. doi: 10.1177/1535759720928269
